# Chinese Herbal Formula, Modified Danggui Buxue Tang, Attenuates Apoptosis of Hematopoietic Stem Cells in Immune-Mediated Aplastic Anemia Mouse Model

**DOI:** 10.1155/2017/9786972

**Published:** 2017-08-16

**Authors:** Jingwei Zhou, Xue Li, Peiying Deng, Yi Wei, Juan Liu, Meng Chen, Yamei Xu, Dongmei Zhang, Lingqun Zhu, Lixia Lou, Bin Dong, Qiushuo Jin, Limin Chai

**Affiliations:** ^1^Key Laboratory of Chinese Internal Medicine of Ministry of Education and Beijing, Dongzhimen Hospital, Beijing University of Chinese Medicine, Beijing 100700, China; ^2^Department of Rheumatology, Dongzhimen Hospital, Beijing University of Chinese Medicine, Beijing 100700, China; ^3^School of Preclinical Medicine, Beijing University of Chinese Medicine, Beijing 100029, China; ^4^Department of Hematology & Oncology, Dongzhimen Hospital, Beijing University of Chinese Medicine, Beijing 100700, China

## Abstract

A derivative formula, DGBX, which is composed of three herbs (*Radix astragali*, *Radix Angelicae sinensis*, and *Coptis chinensis* Franch), is derived from a famous Chinese herbal formula, Danggui Buxue Tang (DBT) (*Radix astragali* and *Radix Angelicae sinensis*). We aimed to investigate the effects of DGBX on the regulation of the balance between proliferation and apoptosis of hematopoietic stem cells (HSCs) due to the aberrant immune response in a mouse model of aplastic anemia (AA). Cyclosporine (CsA), an immunosuppressor, was used as the positive control. Our results indicated that DGBX could downregulate the production of IFN*γ* in bone marrow cells by interfering with the binding between SLAM and SAP and the expressions of Fyn and T-bet. This herbal formula can also inhibit the activation of Fas-mediated apoptosis, interferon regulatory factor-1-induced JAK/Stat, and eukaryotic initiation factor 2 signaling pathways and thereby induce proliferation and attenuate apoptosis of HSCs. In conclusion, DGBX can relieve the immune-mediated destruction of HSCs, repair hematopoietic failure, and recover the hematopoietic function of HSCs in hematogenesis. Therefore, DGBX can be used in traditional medicine against AA as a complementary and alternative immunosuppressive therapeutic formula.

## 1. Introduction

Aplastic anemia (AA) is a rare acquired bone marrow failure syndrome resulting from immune-mediated destruction of hematopoietic stem cells (HSCs), caused largely by many quantitative and qualitative defects in HSCs [1]. Allogeneic stem cell transplant can cure severe AA. Nevertheless, transplantation in patients who are old or lack family donors has many challenges [2]. The molecular basis of the aberrant immune response and deficiencies in HSC is now being defined genetically. Immunosuppression by antithymocyte globulins and cyclosporine A (CsA) is effective on restoring blood cell production in the majority of AA patients. However, the incidence of relapse and evolution of clonal hematological diseases remain high [3].

Danggui Buxue Tang (DBT), one of the simplest traditional Chinese medicine (TCM) decoctions, has been used in the treatment of blood deficiency syndrome for more than 800 years in China. DBT is composed of two herbs, *Radix astragali* (Milkvetch Root) and *Radix Angelicae sinensis* (Chinese Angelica), boiled together at a 5 : 1 ratio. Pharmacological studies indicated that DBT has effects on bone development [4], blood enhancement [5], and immune stimulation [6]. Several studies have verified the hematopoietic function of DBT in a mouse model of bone marrow suppression. It can increase the production of peripheral blood and nucleated bone marrow cells (BMCs) and the colony count of hematopoietic stem/progenitor cells (HSPCs) *in vitro*; regulate the expression of cytokines, such as erythropoietin, thrombopoietin, and granulocyte-macrophage colony-stimulating factor in bone marrow microenvironment; and contribute to cell cycle of HSPCs [7]. For the bone marrow failure syndrome resulting from immune-mediated destruction of HSC, we modified DBT to prepare a derivative herb formula (DGBX) by adding *Coptis chinensis* Franch (Coptidis rhizoma) for reinforcing the regulatory effect on aberrant immune response in the bone marrow of patients with AA. Pharmacological studies validated that *Coptis chinensis* Franch modulates immune responses and controls certain inflammation-related diseases [8]. The combination of these three herbs can contribute to multitargeting, multichannel immunosuppressive effects in AA treatment.

We investigated the possible mechanisms underlying the immunosuppressive and hematopoietic functions of DGBX in an immune-mediated AA mouse model. CsA was used as a positive control. The aim of this study was to identify the specific cellular targets involved in the immunosuppressive and hematopoietic functions of DGBX in AA treatment.

## 2. Materials and Methods

### 2.1. Preparation of Herbal Composition of DGBX

*Radix astragali*, *Radix Angelicae sinensis*, and *Coptis chinensis* Franch (total weight, 126 g; individual ratio, 5 : 1 : 1) were boiled together in 6× volume of water for 0.5 h. Then, residue from the first extraction was boiled in 8× volume of water for 25 min. Finally, the filtered solutions were combined and concentrated into aqueous extracts containing 0.9 g/mL of raw herbs.

### 2.2. High-Performance Liquid Chromatography-Electrospray Ionization/Mass Spectrometry

The herbal extracts were filtered using a 150 *μ*m standard test sieve and maintained in desiccators at 4°C until use. Lyophilized flower (0.02 g) was extracted with 5 mL of methanol/water (*v*/*v* = 1 : 1) in the sonicator for 20 min at room temperature. The extract was filtered through a 0.22 *μ*m membrane. A 10 *μ*L aliquot of the extract was injected into the analytical column for analysis. The compounds were separated on a Phenomenex Kinetex C18 (2.6 *μ*m, 100 × 2.1 mm) operated at 35°C. The mobile phase (0.1% formic acid in water (A) and acetonitrile (B) as mobile phase) was delivered at a flow rate of 0.4 mL/min under a gradient program. The diode-array detector was set at 254 nm, and the online UV spectra were recorded in the scanning range of 190–400 nm.

The optimized parameters for negative and positive modes were as follows: the ion spray voltage was set at 5500 (positive ion mode) and −4500 V (negative ion mode); the turbo V spray temperature at 600°C, nebulizer gas (gas 1) at 50 psi, heater gas (gas 2) at 60 psi, collision gas at medium, the curtain gas at 30 psi, and the declustering potential at 80 (positive ion mode) and −80 V (negative ion mode). The collision energy was set at 35 (positive ion mode) and −35 V (negative ion mode), and the collision energy spread was set at 15 V for MS/MS experiments. The data was acquired with IDA (information-dependent acquisition) method and analyzed by Peak View Software™ 2.2 (SCIEX, Foster City, CA, USA).

### 2.3. Mice

BALB/c female mice (*n* = 6 per group, 7-8 weeks old) and DBA/2 female mice (7 to 8 weeks old) were purchased from HFK Bioscience Co. Ltd. (Beijing, China). Animal care and use were in accordance with the institutional guidelines. All animal experiments were approved by the Institutional Animal Care and Use Committee of the National Institute of State Scientific and Technological Commission.

### 2.4. Induction AA

BALB/c mice, except mice in the normal group, received a sublethal total body irradiation dose of 3.5 Gy from Model 143 ^137^Cesium *γ*-irradiator one hour before lymph node cell infusion. Inguinal, brachial, and axillary infusion of lymph node cells were obtained from female DBA/2 mice. Lymph cells (1 × 10^6^) were injected into BALB/c mice through the tail vein for inducing AA [9].

### 2.5. Drug Treatment

The treatment was initiated after lymph node cell infusion and lasted for 28 days. Mice were randomly divided into four groups: normal group, mice were fed the control diet and orally administered sterile saline; model group, mice were fed the same as the normal group; cyclosporine (CsA) group, CsA (batch number H10960122, Zhongmei Huadong Pharmaceutical Co. Ltd., Hangzhou, China) mice were fed the same control diet and orally daily administered 25 mg/kg CsA daily for 28 days; and DGBX group, mice were fed the same control diet and orally administered 6.3 g/kg DGBX daily for 28 days. The mice were sacrificed on the 29th day after treatment. Mice were anesthetized by isoflurane anesthesia (2-3% isoflurane with oxygen supply). Peripheral blood samples were collected by removing eyeballs, and BMCs were obtained by femoral cavity flushing.

### 2.6. Enzyme-Linked Immunosorbent Assay

Twenty-four hours after the last administration, 0.8 mL of peripheral blood was collected from each mouse by eyeball extirpation. Sera were isolated by centrifuging at 3000 rpm and 4°C for 10 min. The concentrations of interferon *γ* (IFN*γ*), interleukin-2 (IL-2), and tumor necrosis factor *α* (TNF*α*) were measured using ELISA kits (eBioscience, San Diego, CA, USA), according to the instructions provided by the manufacturer.

### 2.7. Fluorescence-Activated Cell Sorter Analysis

BMCs were obtained by femoral cavity flushing and filtered through a 200-eye cell sieve mesh to obtain a single-cell suspension. To quantify the percentage of HSCs, CD117- and sac-1-positive cells were washed and stained with anti-mouse CD117 (c-Kit) FITC and anti-mouse Ly-6A/E (Sca-1) PE antibodies (eBioscience). For the detection of apoptosis in BMCs, an Annexin V-FITC Apoptosis Detection Kit (eBioscience) was used. BMCs were stained with FITC-conjugated annexin V and propidium iodide (PI). Flow cytometry was performed using a fluorescence-activated cell sorter Calibur cytometer and analyzed with CellQuest software (Beckman Coulter, Brea, CA, USA).

### 2.8. Immunofluorescence

To examine the expression and distribution of T-bet in BMCs, cells were fixed, permeabilized, and incubated with anti-human/mouse T-bet PE (1 : 100) (eBioscience). To identify changes in binding of signaling lymphocyte activation molecule (SLAM) and SLAM-associated protein (SAP), cells were stained with anti-mouse CD150/SLAM PE antibody (1 : 50) (eBioscience). After permeabilization, cells were incubated with SH2D1A/SAP antibody (FITC) (1 : 100) (EterLife, Birmingham, UK). Confocal fluorescence microscopy images were captured using a Leitz/Leica TCSSP2 microscope (Leica Lasertechnik GmbH, Heidelberg, Germany). The fluorescence intensity was quantified with ImageJ. At least 50 cells from 3 different areas of each chamber were measured.

### 2.9. Western Blot Analysis

BMCs (1 × 10^7^) from each mouse were lysed in 0.5 mL of lysis buffer (Sigma, St. Louis, MO, USA). The extracts were cleared by centrifuging at 10,000*g* and 4°C for 15 min and diluted with the lysis buffer to achieve about 2 mg/mL protein concentration. Protein samples were separated on 10% SDS-PAGE and transferred onto nitrocellulose membranes (Amersham Pharmacia Biotech, Uppsala, Sweden). The membranes were incubated with primary antibodies, including anti-T-bet/Tbx21antibody (1 : 1000), anti-Fas antibody (1 : 1000) (Abcam, Cambridge, MA, USA), and anti-mouse-caspase-3, anti-mouse-cleaved caspase-3 antibody, anti-mouse-eukaryotic initiation factor 2 (eIF2) *α* (D7D3), anti-mouse-phospho-eIF2*α* (Ser51) (D9G8), anti-mouse-Fyn, anti-mouse interferon regulatory factor-1 (IRF-1), anti-mouse-signal transducer and activator of transcription (Stat) 1, and anti-mouse-Stat3 rabbit monoclonal antibodies (1 : 1000, CST, Boston, MA, USA) and incubated with horseradish peroxidase-conjugated secondary antibody (CST). All immunoreactive proteins were visualized with SuperSignal West Pico Chemiluminescent Substrate (Thermo Scientific, Rockford, IL, USA). Densitometry plots showing protein expression were normalized to GAPDH.

### 2.10. Statistical Analysis

All data were presented as mean ± standard deviation (S.D.). The statistical analyses were performed using SPSS13.0 (SPSS Inc., Chicago, USA). One-way analyses of variance (ANOVA) followed by the Tukey-Kramer test for multiple comparisons were used to compare the treatment groups. A *P* value of <0.05 was considered statistically significant.

## 3. Results

### 3.1. Characteristics of Pure Compounds from the Herbal Formula DGBX

**Figure 1:**
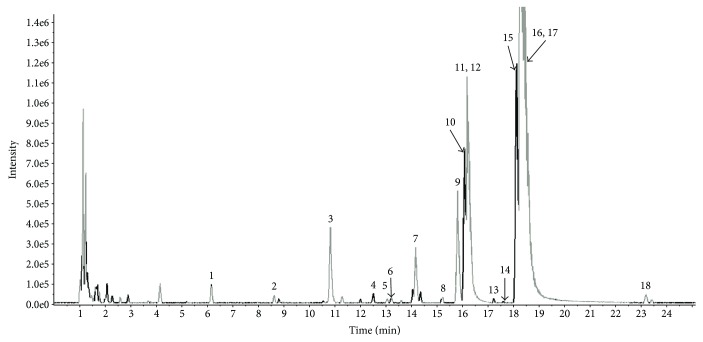
High-performance liquid chromatography-electrospray ionization/mass spectrometry ion chromatograms of lyophilized boiled aqueous extract of DGBX. Abscissa represents retention time, and ordinate represents chromatographic peak intensity.

In this study, high-resolution MS was performed in negative and positive ion modes to obtain complete information about the chemical constitution of DGBX. The peak MS spectrum has been presented in Figure 1. Eighteen constituents were identified based on the accurate mass and relative ion abundance of the target peaks. The identified compounds are shown in Table 1.

### 3.2. Effects of DGBX on Proliferation and Apoptosis of HSCs in AA Mouse Model

**Figure 2:**
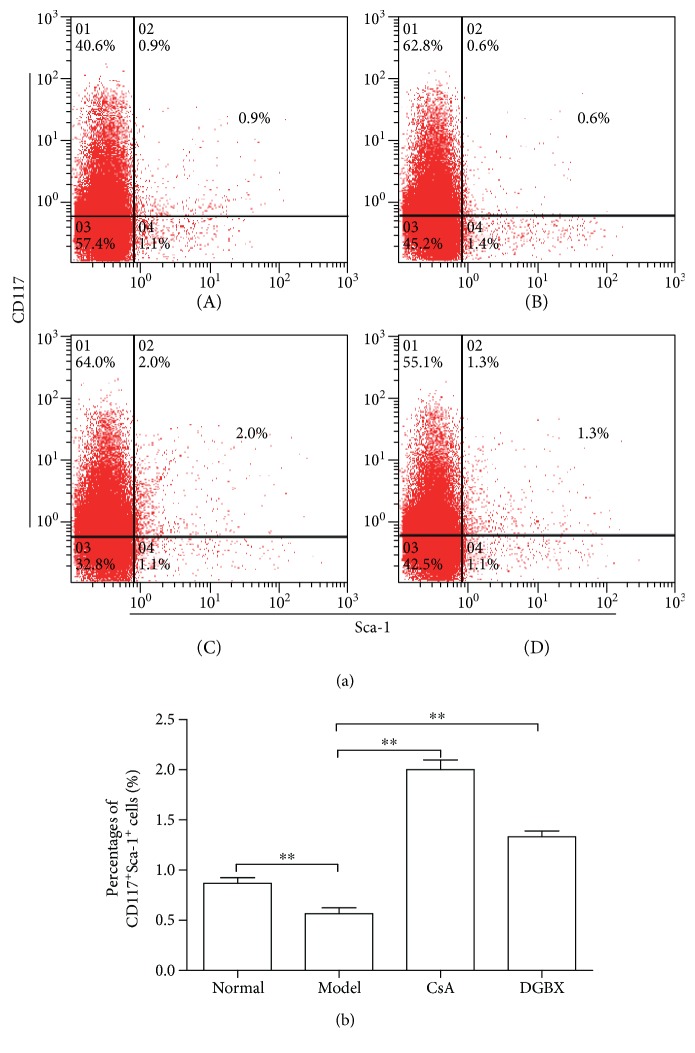
Effects of DGBX on proliferation of hematopoietic stem cells in bone marrow of mice with aplastic anemia (AA). (a) The percentage of CD117+Sca-1+ hematopoietic stem cells in the bone marrow cells of mice after 4 weeks of treatment. A, normal group, B, model group, C, group treated with cyclosporine A, and D, group treated with DGBX. (b) Results are presented in the bar charts. Data are presented as mean ± SD, *n* = 6. ^∗∗^*P* < 0.01.

Sca-1 and c-kit (CD117) are the major phenotypic markers for mouse HSPC subset [10]. Therefore, we quantified CD117^+^Sca-1^+^ cells to assess the percentages of HSCs in total BMCs. As shown in Figure 2, the amount of HSCs in model group significantly decreased compared with that in normal group (*P* < 0.01). HSC proliferation was significantly induced in DGBX group compared with that in the model group (*P* < 0.01).

**Figure 3:**
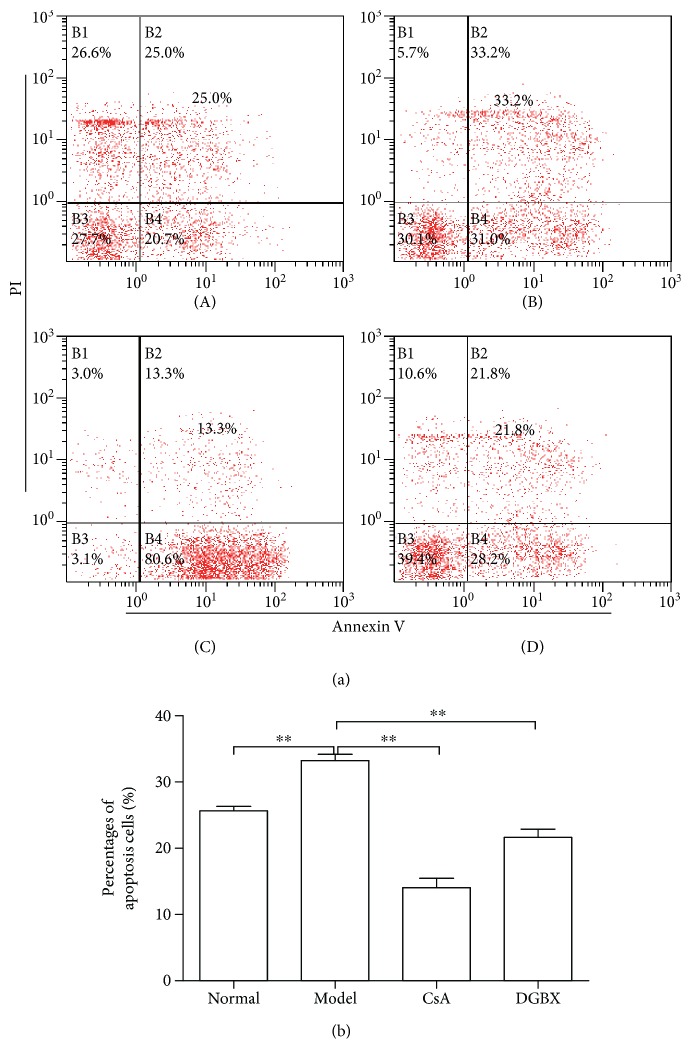
DGBX suppresses apoptosis of bone morrow cells in mice with aplastic anemia (AA). (a) The percentages of annexin V^+^ PI^+^ cells in bone marrow of mice after 4 weeks of treatment. A, normal group, B, model group, C, group treated with cyclosporin A, and D, group treated with DGBX. (b) Results are presented in the bar charts. Data are presented as mean ± SD, *n* = 6. ^∗∗^*P* < 0.01.

We also observed the percentage of apoptosis in BMCs by AnnexinV-PI staining using a flow cytometer. The apoptosis ratio of BMCs in model group was significantly higher than that in normal group (*P* < 0.01). DGBX significantly inhibited the apoptosis of BMCs; apoptosis ratio in DGBX group significantly lower than that in model group (*P* < 0.01) (Figure 3).

### 3.3. Inhibition of IFN*γ*, TNF*α*, and IL-2 Production in AA Mice by DGBX

**Figure 4:**
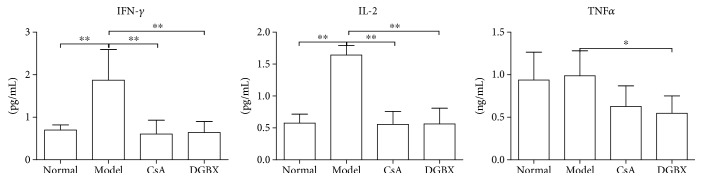
Evaluation of interferon *γ* (IFN*γ*), interleukin-2 (IL-2), and tumor necrosis factor *α* (TNF*α*) levels in sera of mice with aplastic anemia (AA) after treatment by using enzyme-linked immunosorbent assay. The results are presented in the bar chart. Data are presented as mean ± SD, *n* = 6. ^∗^*P* < 0.05, ^∗∗^*P* < 0.01.

As shown in Figure 4, the levels of IFN*γ* and IL-2 in model group were significantly higher than those in normal group (*P* < 0.01), and there was no significant difference in TNF*α* levels between the groups. After treatment with CsA and DGBX, the abnormally high levels of IFN*γ* and IL-2 significantly decreased (*P* < 0.01) and the levels of TNF*α* also significantly decreased compared with that in the model group (*P* < 0.05). These results indicated that DGBX treatment could inhibit the production of inflammatory factors and that the inhibitory effect of DGBX was equivalent to that of CsA.

### 3.4. Effects of DGBX on Activation of SLAM/SAP Signaling Pathway

**Figure 5:**
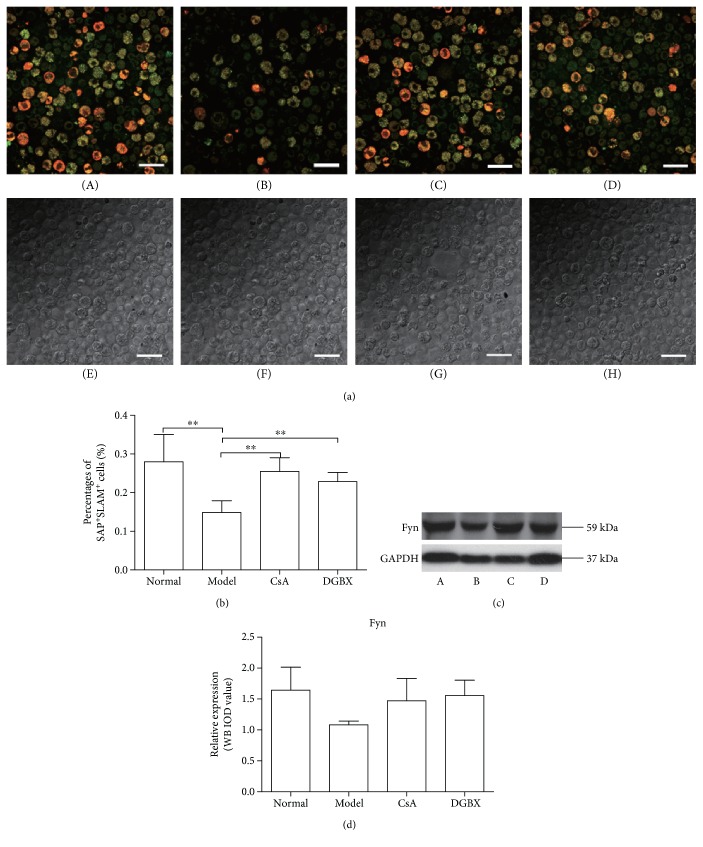
DGBX attenuates interferon *γ* (IFN*γ*) expression by regulating the activation of SLAM/SAP signal. (a) BMCs were stained for CD150/SLAM (red) and SH2D1A/SAP (green) antibodies and observed by confocal immunofluorescence microscopy. The bottom pictures were obtained in bright field. The double-stained cells (yellow) were quantified with ImageJ. (b) Results are presented in the bar charts. The scale bar corresponds to 100 *μ*m throughout. A, normal group, B, model group, C, group treated with cyclosporin A, and D, group treated with DGBX. (c) Fyn was assessed in whole BMC lysates by Western blot analysis. (d) The results are presented in the bar chart. GAPDH was used as an internal control. Data are presented as mean ± SD, *n* = 6. ^∗∗^*P* < 0.01.

The SLAM-SAP signaling is an important pathway in T cell activation when engaged with ligands of T cell receptor [11]. SAP/SLAM recruits Fyn (a member of the Src family of kinases) that inhibits the expression of IFN*γ* [12]. As shown in Figure 5, immunofluorescence analysis showed that the SLAM/SAP double-stained cells in the bone marrow of AA mice significantly decreased compared with that in normal group (*P* < 0.01) and the decline was significantly inhibited by DGBX treatment (*P* < 0.01) (Figure 5). However, DGBX treatment had no effect on the protein expression of Fyn.

### 3.5. DGBX Interferes with T-bet Expression and Distribution in AA Mice

**Figure 6:**
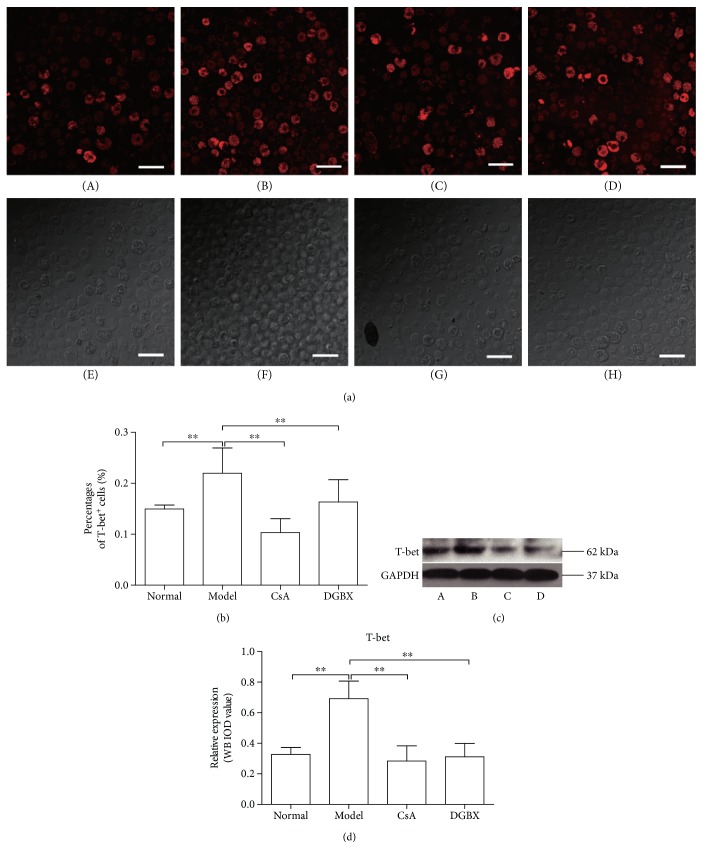
Expression and distribution of T-bet in bone marrow cells (BMCs) of mice with aplastic anemia (AA) after treatment. (a) Staining for T-bet (red) in BMCs was observed by confocal immunofluorescence microscopy. The bottom pictures were obtained in bright field. The stained cells (red) were quantitated with ImageJ. (b) Results are presented in the bar charts. The scale bar corresponds to 100 *μ*m throughout. A, normal group, B, model group, C, group treated with cyclosporin A, and D, group treated with DGBX. (c) The expression of T-bet was evaluated in whole BMC lysates by Western blot analysis. (d) The results are presented in the bar chart. Data are presented as mean ± SD, *n* = 6. ^∗∗^*P* < 0.01.

T-bet, a transcription factor, binds to the IFN*γ* promoter region and induces gene expression [13]. As shown in Figure 6, the T-bet^+^-stained cells significantly increased in bone marrow of AA mice (*P* < 0.01) and T-bet expression significantly increased (*P* < 0.01) compared with that in normal group. DGBX treatment significantly inhibited the levels of T-bet^+^ cells and protein expression. Furthermore, the regulatory effect of DGBX was equivalent to CsA.

### 3.6. Effects of DGBX on Activation of Eukaryotic Initiation Factor 2*α* in BMCs of AA Mice

**Figure 7:**
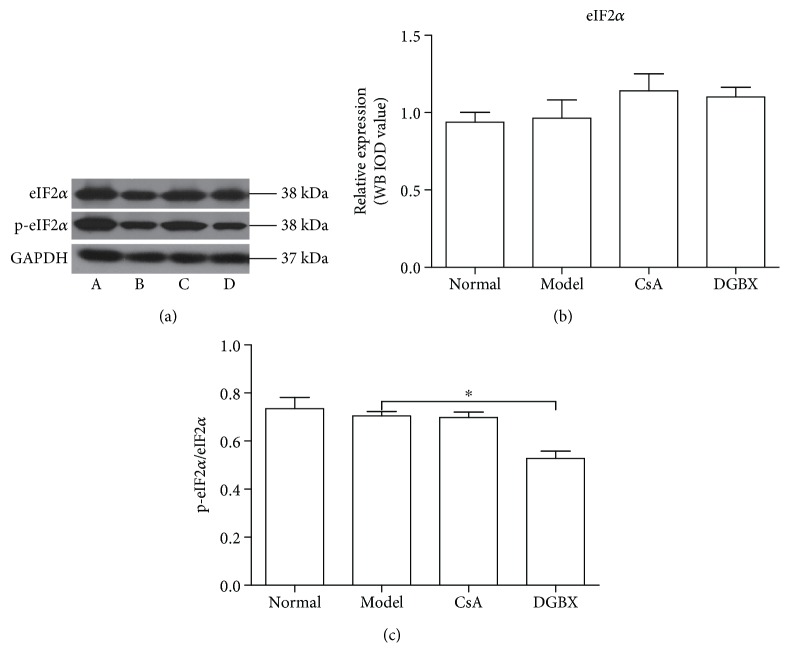
Effects of DGBX on phosphorylation of eIF2*α* in bone marrow cells (BMCs) of mice with aplastic anemia (AA). (a) The expressive levels of eIF2*α* and phospho-eIF2*α* were estimated in whole BMC lysates by Western blot analysis. (b) Results are presented in the bar charts. A, normal group, B, model group, C, group treated with cylosporin A, and D, group treated with DGBX. (c) The ratio of phospho-eIF2*α* and total eIF2*α* is presented in the bar chart. GAPDH was used as the internal control. Data are presented as mean ± SD, *n* = 6. ^∗^*P* < 0.05.

Phosphorylation of the *α*-subunit of eIF2 is a well-documented mechanism of downregulation of protein synthesis under various stress conditions. Western blot analysis demonstrated that eIF2*α* expression in BMCs was not different between the groups. The phosphorylation level of total eIF2*α* in DGBX group showed a decreasing trend compared with that in the model group (*P* < 0.05) (Figure 7).

### 3.7. DGBX Regulates Expression of Key Molecules of Fas-Mediated Apoptosis Signaling Pathway in BMCs of AA Mice

**Figure 8:**
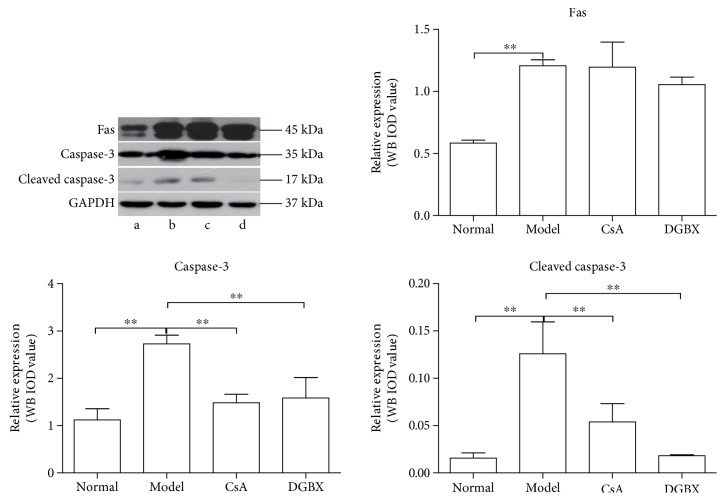
Effect of DGBX on the expressions of key molecule of Fas-dependent apoptosis pathway in AA mice. The protein levels of Fas, caspase-3, and cleave caspase-3 were estimated in whole BMC lysates by Western blot analysis. Results are presented in the bar charts. Data are presented as means ± SD, *n* = 6. ^∗∗^*P* < 0.01.

As shown in Figure 8, the levels of Fas, caspase-3, and cleave caspase-3 in model group were significantly higher than those in normal group (*P* < 0.01). DGBX significantly downregulated the expression of caspase-3 and cleaved caspase-3 compared with that in the model group (*P* < 0.05 or *P* < 0.01). Interestingly, the regulatory effects of DGBX were superior to those of CsA. However, DGBX did not exert a regulatory effect on the expression of Fas in BMCs of AA mice.

### 3.8. DGBX Regulates Expression of Interferon Regulatory Factor 1 and Signal Transducer and Activator of Transcription 1 and 3 (Stat1 and Stat3) in BMC of AA Mice

**Figure 9:**
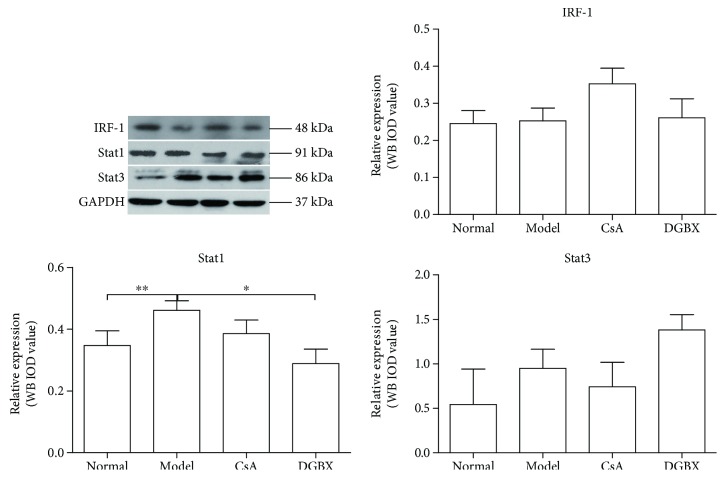
Effects of DGBX on regulation of expression of key molecules of interferon regulatory factor-1-mediated JAK/Stat signaling pathway in mice with aplastic anemia (AA). IRF-1, Stat1, and Stat3 expressions were estimated in whole BMC lysates by Western blot analysis. Data are presented as mean ± SD, *n* = 6. ^∗^*P* < 0.05, ^∗∗^*P* < 0.01.

IRFs play an important role in the defense against pathogens, autoimmunity, lymphocyte development, cell growth, and susceptibility to transformation. Some effects of IFN*γ* are mediated through IRF-1, which inhibits the transcription of cellular genes and entry into the cell cycle. Stat1 and Stat3 contribute to this function [12]. The protein levels of Stat1 in the model group were significantly higher than those in AA mice (*P* < 0.01). DGBX downregulated the abnormally high expression of Stat1 (*P* < 0.05), but had no regulatory effect on the expression of IRF-1 (Figure 9).

## 4. Discussion

In TCM, combinatory therapeutic strategies based on patient symptoms and characteristics are often adopted to treat several diseases [14]. The Chinese herb formulae consist of several types of medicinal herbs or minerals, and multiple components could act on multiple targets and exert synergistic therapeutic efficacies [15]. Recently, LC-MS becomes an essential tool for analyzing the compounds of the herbal constituents in TCM complex formulae [16]. Eighteen constituents were identified by high-performance liquid chromatography-electrospray ionization/mass spectrometry from freeze-dried boiled aqueous extract of DGBX. The major components of DGBX-lyophilized powder were identified to be magnoflorine (3), tetradehydroscoulerine (7), jatrorrhizine (9), ononin (10), epiberberine (11), coptisine (12), palmatine (15), berberine (16), and calycosin (17) by Peak View Software analysis. Magnoflorine exhibits immunomodulatory effects on neutrophil and T cell-mediated immunity [17]. Coptisine can inhibit IL-1*β*-induced inflammatory response by suppressing the nuclear factor-kappa B (NF-*κ*B) signaling pathway [18]. Jatrorrhizine plays a critical cell-protective role in H_2_O_2_-induced cell apoptosis owing to its antioxidative property [19]. Palmatine plays an important role in osteoclast apoptosis by regulating the inducible nitric monoxide synthase (iNOS) system [20]. Berberine can also decrease cell apoptosis induced by LPS by suppressing iNOS protein expression [21]. Calycosin reduces oxidative stress by regulating the activation of the PI3K/Akt/GSK-3*β* signaling pathway and suppressing osteoclastogenesis through inhibition of MAPK and NF-*κ*B activation [22]. AA is an acquired bone marrow failure syndrome resulting from immune-mediated destruction of HSC. Therefore, we suggest that the functions of these herbal constituents of DGBX contribute to interaction with multiple targets and exert synergistic therapeutic efficacy in immunosuppression and hematopoiesis against AA.

Dysregulation in HSC cycling contributes to AA by enhancing differentiation over self-renewal or inducing apoptosis of HSCs [23]. Acquired AA exhibits increased levels of circulating IFN*γ*. IFNs have the pathogenic functions of impairing the proliferation of primitive HSPCs [24] and inducing apoptosis [25]. These evidences support that IFNs can impair hematopoiesis by attenuating HSC function. Antigen-presenting cells present antigens to T lymphocytes and trigger T cells to proliferate. The prototypical Th1 transcription factor T-bet binds to the IFN*γ* gene promoter region and induces gene expression [26]. CD150/SLAM is a prototypical member of glycoprotein receptors on hematopoietic cells. It is unique in its binding to the signaling molecule SAP [27]. The binding of SAP and Fyn modulates the SLAM function in IFN*γ* expression and then decreases IFN*γ* gene transcription [12]. Constitutive T-bet expression and low SAP levels are observed in AA. Activated T cells also produce proinflammatory cytokine at abnormal levels. Increased production of IL-2 leads to polyclonal expansion of T cells. These immune responses initiate the immune-mediated T cell destruction of BMCs. We observed that the levels of circulating IFN*γ*, TNF*α*, and IL-2 in AA mice decreased to levels similar to those of normal group after treatment with DGBX. The quantity of SLAM/SAP double-stained cells significantly decreased, and the expression of Fyn was suppressed in BMCs by DGBX treatment. In addition, DGBX could inhibit the expression of T-bet, decreasing the level of T-bet-positive cells in the bone marrow. We believe that the immunosuppressive mechanisms of DGBX include SLAM-mediated T cell proliferation and IFN*γ* production for attenuating the immune-mediated destruction of bone marrow.

In the pathogenesis of the immune destruction of hematopoiesis, aberrant immune response induced IFN*γ* production as well as TNF*α* and IL-2. IFN*γ* and TNF*α* activate the T cell cellular receptors and Fas receptor [28]. Trimerization of Fas receptor interacts with Fas-associated death domain, activates caspase-3, and ultimately promotes HSC apoptosis [29]. IFN*γ* can also activate the cell signaling cascade of IRF-1, contributing to the inhibition of the transcription of cellular genes and induction of eIF-2 phosphorylation for suppressing protein synthesis. IFN*γ* induces IRF-1 binding to IFN-stimulated response elements, subsequently activating Stat1 and Stat3, inducing gene transcription associated with inflammation, and contributing to the immune-mediated destruction of the bone marrow [30]. This pathophysiology results in hematopoietic failure in AA. In this study, we found that the expression of phosphor-eIF2*α*, Stat1, Stat3, and caspase-3 significantly decreased in AA mice treated with DGBX. There were no regulatory effects of DGBX on the expression of Fas and IRF-1. These results indicated that DGBX could inhibit the aberrant immune response and the apoptosis of HSCs by modulating the activation of Stat/JAK/IRF-1 pathway and Fas-dependent pathway.

CsA is a calcineurin inhibitor. It has selective effect on T cell functions by direct inhibition of the expression of nuclear regulatory proteins, contributing to reduction of T cell proliferation and activation. Severe AA can respond to CsA alone. Allogeneic bone transplantation and immunosuppressive treatment with antithymocyte globulin and CsA can significantly increase the 10-year survival rate in patients with severe AA [31]. In this study, we also assessed the significant effects of CsA on the inhibition of proinflammatory cytokine and related protein expression, which participate in the immune response. CsA has many side effects, such as kidney damage and hepatotoxicity. These side effects can be managed by dose reduction [12]. Multiherbal formulas based on traditional medicine have been scientifically verified for use in complementary and alternative therapy for various diseases. Formulae composed of a mixture of natural products can target multiple sites. In our study, DGBX was verified as an effective medicine for AA treatment. It can also avoid kidney damage, hepatotoxicity, and other adverse reactions of drugs including CsA. Our results indicated that the immunosuppressive effects that aided recovery of the hematopoietic function of HSCs in hematogenesis in AA mice were equivalent or exceeded those of CsA.

## 5. Conclusions

Our data indicated that DGBX could attenuate IFN*γ* production by interfering in SLAM/SAP signaling and production and distribution of T-bet in T cells, exert immunosuppressive effects by modulating the activation of the Stat/JAK/IRF-1 pathway, restrain cell apoptosis by intervening in Fas-dependent pathway, and eventually attenuate immune-mediated destruction of HSCs, repair hematopoietic failure, and recover the hematopoietic function of HSCs in hematogenesis for AA therapy. DGBX can be used in traditional medicine against AA as a complementary and alternative immunosuppressive therapeutic formula.

## Figures and Tables

**Table 1:** Chemical components identified from DGBX by high-performance liquid chromatography-electrospray ionization/mass spectrometry.

Peak	*t*_R_ (min)	Formula	Identification
1	6.14	C_11_H_12_N_2_O_2_	L-tryptophan
2	8.61	C_17_H_20_O_9_	3-O-Feruloylquinic
3	10.82	C_20_H_24_NO_4_	Magnoflorine
4	12.5	C_22_H_22_NO_10_	Calycosin-7-O-*β*-D-glucoside
5	13.04	C_10_H_10_O_4_	Ferulic acid
6	13.2	C_12_H_16_O_4_	Senkyunolide I
7	14.17	C_22_H_24_NO_4_^+^Cl^−^	Tetradehydroscoulerine
8	15.22	C_22_H_22_NO_10_	Calycosin-7-O-*β*-D-glucoside-6″-O-malonate
9	15.81	C_20_H_20_NO_4_	Jatrorrhizine
10	16.01	C_22_H_22_O_9_	Ononin
11	16.08	C_20_H_18_NO_4_	Epiberberine
12	16.21	C_19_H_14_NO_4_	Coptisine
13	16.67	C_24_H_26_O_10_	(6aR,-11aR)-3-Hydroxy-9,10-dimethoxypterocarpan-3-O-*β*-D-glucoside
14	17.6	C_12_H_12_O_2_	Z-Butylidenephthalide
15	18.12	C_21_H_25_NO_4_	Palmatine
16	18.31	C_20_H_18_NO_4_	Berberine
17	18.47	C_16_H_12_O_5_	Calycosin
18	23.19	C_16_H_12_O_4_	Formononetin
